# Rethinking Responsive Feeding: Insights from Bangladesh

**DOI:** 10.3390/nu14153156

**Published:** 2022-07-30

**Authors:** Maureen M. Black, Fahmida Tofail, Eric A. Hodges, Carla M. Bann, Jena D. Hamadani, Shirina Aktar, Chessa K. Lutter

**Affiliations:** 1 RTI International, Research Triangle Park, NC 27709, USA or maureenblack@rti.org (M.M.B.); cmb@rti.org (C.M.B.); 2 Department of Pediatrics, University of Maryland School of Medicine, Baltimore, MD 21201, USA; 3 International Centre for Diarrhoeal Disease Research, Bangladesh (icddr,b), Dhaka 1000, Bangladesh; ftofail@icddrb.org (F.T.); jena@icddrb.org (J.D.H.); shirina.aktar@icddrb.org (S.A.); 4 Chapel Hill School of Nursing, University of North Carolina, Chapel Hill, NC 27599, USA; eric.a.hodges@unc.edu

**Keywords:** responsive feeding, infants, complementary feeding, mother-infant observation

## Abstract

Young children’s growth is influenced by food and feeding behavior. Responsive feeding has been shown to promote healthy growth and development, to prevent under- and overfeeding, and to encourage children’s self-regulation. However, most measures of responsive feeding do not incorporate bidirectional mother-infant responsivity or early learning principles and have not been validated against observations. To overcome these gaps, we laid the groundwork for a responsive feeding measure based on a community sample of 67 mothers and their 6–18-month-old children in Bangladesh. Children were weighed and measured. Mothers reported on their child’s dietary intake and responded to a 38-item responsive feeding questionnaire developed through a 2-phase Delphi procedure. Based on a video-recorded feeding observation, mother-child dyads were categorized into proximal (43%) and distal (57%) responsivity groups. Using stepwise logistic regression, a 9-item model from the responsive feeding questionnaire had excellent fit (AUC = 0.93), sensitivity (90%), specificity (89%), positive predictive value (87%), and negative predictive value (93%). Proximal responsivity was characterized by maternal concerns about children’s dietary intake. Distal responsivity was characterized by maternal perception of children’s happy mood during feeding. Findings support responsive feeding as modulating between proximal and distal responsivity, promoting autonomy, self-regulation, and enabling children to acquire and practice healthy eating behaviors.

## 1. Introduction

Throughout the world, at least 1 in 3 children under 5 years of age is experiencing stunting, wasting, or overweight [1]. These early growth problems increase children’s risk for long term problems in health, academics, and well-being. Global guidance recognizes that both what children are fed and how they are fed are important aspects of infant and young child feeding [2].

Feeding infants and young children is a bidirectional process with children signaling hunger or satiety through gestures and facial expressions, caregivers recognizing and responding with age- and culturally appropriate feeding behavior, and children experiencing the caregiver’s response in a context of nurturance and emotional comfort [3]. This pattern, known as responsive feeding, is widely recognized [4]. Responsive feeding has been shown to promote healthy growth and development, to prevent both under- and overfeeding, and to encourage children’s self-regulation, an important precursor for the prevention of both undernutrition and overweight [3,5,6]. As such, responsive feeding is relevant globally—in both high income and low- and middle-income countries and settings [5,7]. 

Although responsive feeding is a critical component of the Nurturing Care Framework, included as a recommendation in WHO’s guidance on improving early childhood development, and is one of 10 Guiding Principles developed by the Pan American Health Organization and the World Health Organization (WHO) for complementary feeding [8,9,10,11], there are no global measures for responsive feeding. With the exception of responsive feeding measures developed in Cambodia and Sri Lanka [12,13], most responsive feeding measures for children under age 2 years have been developed in high-income countries. Most have not been validated against observations, the gold standard [14], and do not include either the distinctions between proximal and distal responsivity inherent in the principles of early learning [15] or the bidirectional components of responsive feeding [16]: child signals, caregiver response, and nurturant context [3]. These omissions, together with a primary focus on caregiver mealtime behaviors suggest a lack of clarity regarding the conceptual basis of responsive feeding [17]. 

**Figure 1:**
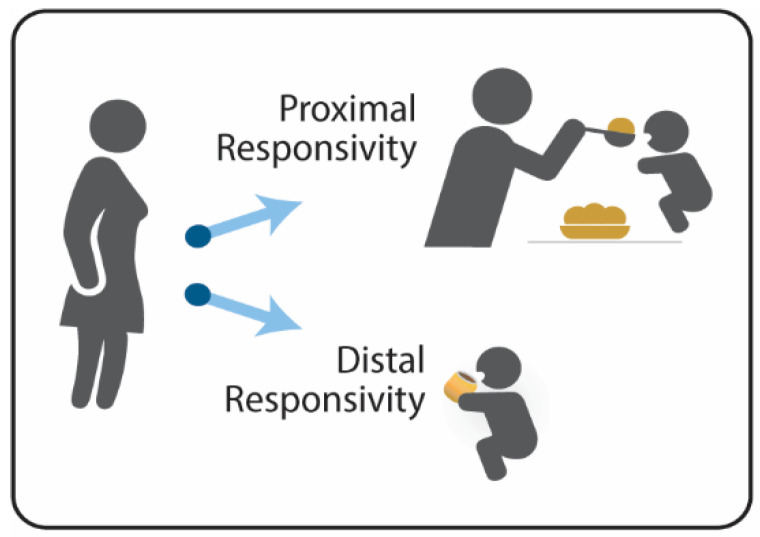
Modulating Between Proximal and Distal Responsivity.

The bidirectional process that underlies responsive feeding is based on principles of early learning and caregiver-child transactions [15,18]. Caregivers modulate their responsivity by providing proximal support (i.e., direct assistance) as children are acquiring skills, such as learning to eat, and distal support (i.e., nods and smiles) as children are practicing, establishing mastery, and building autonomy and self-regulation (Figure 1) [15]. The modulation between proximal and distal responsivity varies as children acquire and practice new skills. By focusing primarily on the presence or absence of proximal responsivity, distal responsivity may be disregarded or misinterpreted as the lack of responsive feeding. Growth faltering and responsive feeding are major concerns during the complementary feeding period (ages 6–18 months) [19], suggesting the relevance of focusing on caregivers’ responses to their child’s signals and behaviors, along with their awareness of their child’s nutritional and developmental needs [20].

Without either a clear conceptual understanding or a consistent method to measure responsive feeding, countries cannot track population-based initiatives to advance responsive feeding, programs promoting responsive feeding cannot be easily evaluated, and children with feeding difficulties cannot be identified for corrective interventions prior to developing growth problems. Our study begins to fill this void by clarifying the conceptual basis of responsive feeding and preparing for the development and validation of a caregiver-report measure of responsive feeding that could be used globally. We focus on mothers and young children in one country and have two objectives: (1) describe commonly reported feeding behaviors and (2) develop an initial measure of responsive feeding and validate it against video-recorded feeding sessions. 

We conducted the study in Bangladesh, a country in south Asia of over 160 million people, including 64 million children (40%). Bangladesh has experienced economic and social success over the past 30 years and is classified by the World Bank as a lower middle-income country. Based on the 2020 Global Nutrition Report, 65% of children aged 0 to 5 months are exclusively breastfed, 31% of children under 5 years of age are stunted and 8% are wasted, illustrating that poverty and early growth remain significant concerns. 

## 2. Materials and Methods

We recruited mothers and their children aged 6 to 18 months from urban areas of Dhaka, Bangladesh, who were receiving solid food. To develop a measure of responsive feeding, we conducted a 2-phase Delphi procedure, a multi-stage process whereby subject matter experts provide feedback to questions, which are refined and returned to the experts for subsequent feedback [21]. First, we identified 15 measures of responsive feeding and feeding interactions for children aged 6 to 18 months and built an item bank of questions. Second, we invited experts in responsive feeding (n = 15) to participate in the Delphi procedure and asked them to rate questions that represented bidirectional feeding interactions and response choices for children in the 6–18-month age group. Third, after two rounds of revisions with experts, we selected 47 questions with the greatest consensus. 

We had two independent bilingual translators from Bangladesh (university graduates) with more than 5 years of translation experience translate the questions into Bengali. A project co-investigator and site-PI synchronized the Bangla-translated versions and developed the first synthesized version of a questionnaire. After field testing the questionnaire on five field-staff members, we made further revisions, simplified the language, and made a second synthesized version that was reviewed and revised by two subject matter experts from Bangladesh. A third bilingual experienced translator, who was not involved with the study, completed a back translation, which was then matched with the original English version. We administered the final field–tested version of the questionnaire to ten Bangladeshi caregivers in the target community for cognitive testing and final modification. We removed 9 additional questions, resulting in a final responsive feeding questionnaire with 38 items. 

### 2.1. Procedure

Our collaborating partner, the International Centre for Diarrhoeal Disease Research, Bangladesh (icddr,b) has a field station and office in Mirpur, an urban community of approximately one million residents in Dhaka, Bangladesh with a literacy rate of 69%, higher than the national average of 49%. Ethical procedures were approved by the icddr,b (Research Protocol #PR19120; Grant # GR-01877) and RTI through a reliance agreement between the icddr,b and RTI International. 

We conducted a door-to-door survey of the community surrounding the field site to identify mothers with children who met the inclusion criteria of age 6–18 months, born at term (based on maternal report), no identified disabilities or chronic conditions that would interfere with feeding or growth, perceived by mothers to be healthy, and had received complementary food. Inclusion criteria for mothers were age 18 or older, primary caregiver, ability to speak and understand Bengali, agreement with study procedures, and interest in participating in the study. We identified 100 eligible mother-child pairs, and purposefully selected 67 to represent equal distribution across 4 age bands: 6–9 months, 10–12 months, 13–15 months, and 16–18 months. 

We initiated the study in 2020 shortly before the COVID-19 pandemic. We followed the guidelines of Bangladesh and the icddr,b by halting and resuming the study as recommended. We developed a protective protocol and procedures for participants and staff. No cases of COVID-19 were detected among study participants or field staff related to data collection. 

Research assistants contacted mothers to remind them of the appointment and to advise them to bring food that the child typically ate. All mothers signed informed consent for themselves and their child at the appointment as per protocol. 

Mothers and children were seated on mats on the floor, as is typical in Bangladesh. We requested that the mothers feed their child during the session whenever the child was hungry. While waiting for the child or mother to signal hunger or mother wanting to feed the child, we administered a set of questionnaires to the mother while the child played. We video recorded the entire session to capture the child’s hunger cues and the mothers’ responses. We stopped the questionnaires when the mother either indicated that she wanted to feed the child or began to assemble the food and resumed after the feeding ended. Following the feeding observation and the administration of questionnaires, we weighed and measured the child. Mothers were compensated with a small toy at the conclusion of the session. 

### 2.2. Measures

Socio-Demographics. The socio-demographic questionnaire included the caregivers’ information, ages of household members, education and employment of the mother and father, marital status, religion, assets, housing, and income-expenditure information. We asked caregivers to report on their financial resources and indicate how frequently their experienced a monthly deficit (always, sometimes, or never) [22]. 

Food Security. We modified the Household Food Insecurity Access Scale (HFIAS), developed by the Food and Agriculture organization (FAO) to measure food security [23]. The mothers responded to nine questions (yes/no) regarding food access problems over the preceding 4 weeks. To reduce respondent burden, we did not address frequency. We used a modified scoring: (1) Food secure: no items affirmed; (2) Mildly food insecure (compromised dietary quality or preferences): items 1, 2, 3, or 4 are affirmed but not items 5–9; (3) Moderately food insecure (eating less): items 5 or 6 are affirmed but not 7, 8, or 9; and (4) Severely food insecure (going hungry): items 7, 8, or 9 are affirmed. 

Infant and Young Child Feeding (IYCF). We used the World Health Organization (WHO)-developed IYCF feeding questionnaire [24] and analyzed the data as recommended [25]. We reported the number of children breastfed and the number who consumed foods from the 8 food groups defined by WHO within the past 24 h. We calculated children’s minimum dietary diversity, defined as eating foods from at least 5 of the 8 food groups in the previous 24 h, and minimum meal frequency, defined as eating at least 2 meals/day for breastfed children aged 6–8 months, at least 3 meals/day for breastfed children aged 9–23 months, and at least 4 meals and 2 milk feeds/day for non-breastfed children 6–23 months. 

Responsive Feeding Questionnaire. We administered the final version of the responsive feeding questionnaire with 38 feeding related items, most scored by a 4-point Likert scale based on frequency (1-almost never, 2-occasionally, 3-most days, 4-almost every day). 

Responsiveness to Child Feeding Cues Scale (RCFCS). The Responsiveness to Child Feeding Cues Scale (RCFCS) is a validated observational coding scheme for infants and toddlers that measures both maternal and child responsivity during feeding [14,17]. The observations incorporate the context by beginning prior to and extending beyond the meal, thereby providing bidirectional behaviors. The RCFCS include codes for 48 feeding behaviors, categorized as early, active, or late hunger/receptiveness or disinterest/fullness cues [14,17]. Caregivers are rated on their feeding responsivity to hunger, receptiveness/disinterest, and fullness, respectively along a 5-point based on their response to children’s feeding cues. The behaviors underlying the ratings are discrete, well-defined and lend replicable objectivity to the ratings [14]. Both caregivers and children are rated on general responsivity to one another during feeding along a 5-point scale using the mean of visual attentiveness, positive expressiveness, negative expressiveness (reverse scored), and physical disposition from the beginning of food preparation until 1 min after the child stops eating. We used the mean general responsivity score from caregivers and children to define proximally (5) to distally responsive (1). The RCFCS has been tested with non-Hispanic White, non-Hispanic Black, and Hispanic mothers in the United States and their children under age 24 months. Interrater reliability among multiple raters has been excellent [14]. 

Video recorded feeding episode. To the extent possible, the team left the immediate area once the mother began to prepare to feed the child. The camera was fully visible, with no operator, to reduce distractions, and continued throughout the session. 

Anthropometry. Mothers undressed their child to a clean diaper. Weight was measured twice to the nearest 0.1 kg and a third time if measures differed by >0.1 kg with a TANITA 1584 Baby Scale (Tanita Corporation, Tokyo, Japan) and averaged. Recumbent length was measured to the nearest 0.5 cm with a measuring board (ShorrBoards, Olney, MD, USA), using a similar protocol. Body mass index (BMI) was calculated as weight (kg)/height (m)^2^. Weight-for-age, weight-for-length, length-for-age, and mid-upper arm circumference (MUAC) were converted to Z-scores (WAZ, WLZ, LAZ, MUACZ, respectively) using WHO Child Growth Standards [26]. Underweight, wasting, and stunting were defined as <−2 WAZ, WLZ, and LAZ, respectively. 

### 2.3. Reliability Testing of Outcome Measures 

We recruited two research assistants (university graduates with MSc) who received extensive training for 5 days. Following training and prior to data collection, we calculated inter-observer reliabilities with the trainer (r > 0.97 for anthropometric measurements, >0.98 for HHFIS and IYCF, and >0.99 for the responsive feeding and socio-demographic questionnaires) on 20 non-study mother-child dyads. We evaluated test–retest reliabilities after a gap of 7–10 days. The reliabilities were good for HHFIS (r~0.89) and the responsive feeding questionnaire (~0.70) and average for IYCF (r~0.53). All data were collected in Tab (CommCare App, Dimagi, Cambridge, MA, USA).

### 2.4. Video Coding

We used a multi-step procedure to train the two research assistants to code the video recordings and establish inter-observer reliability using four cues from the RCFCS: hunger, receptiveness, disinterest and fullness for both mother and child. The developer of the scale (EAH) provided access to RCFCS training materials, coded two Bangladesh pilot videos to be used for reference, and met with the coding team through video for training, clarification, and consensus. We established inter-observer reliability between trainer and research assistants on 10 videos with >90% agreement. We conducted ongoing reliability assessments between the two research assistants and their supervisor throughout the data collection and coding periods to prevent observer drift. Agreements between each research assistant and the supervisor were >88%.

### 2.5. Sample Size Calculation 

Using the PASS 2022 Power Analysis and Sample Size Software (2022). NCSS, LLC. Kaysville, Utah, USA [27], we estimated the sample size needed to assess the diagnostic validity of the responsive feeding questionnaire for identifying mother-child dyads with proximal vs. distal responsivity based on the area under the ROC curve (AUC). A sample size of 62 or more is needed to achieve at least 80% power to detect a significant difference between an AUC of 0.70 or higher and the AUC expected by chance (AUC = 0.50), assuming a *p*-value of 0.05 and an equal distribution between the proximal and distal responsivity groups. 

### 2.6. Statistical Plan

To characterize caregiver-reported feeding behavior among our sample of mothers and 6–18-month-old children in Bangladesh, we stratified caregivers’ responses to the responsive feeding questionnaire by quartile. Higher values and higher quartiles indicate more frequent endorsement of the behavior, greater concern, greater importance, or more confidence, as applicable. 

Video-based general dyadic responsivity scores were computed as the mean of child and maternal ratings on four aspects of responsivity: (1) visual attentiveness, (2) positive expressiveness, (3) negative expressiveness, and (4) physical disposition. The ratings for negative expressiveness were reverse coded. General dyadic responsivity scores ranged from 1 to 5 with high values indicating proximal responsivity and low scores representing distal responsivity. Mother-child dyads were placed into two groups based on general responsivity scores using a median split: proximal responsivity (scores > 3.5) and distal responsivity (score ≤ 3.5). 

To select items from the responsive feeding questionnaire, we used stepwise logistic regression to determine which self-report items best predicted mother-child dyads with video-based distal responsivity. By design, low odds of distal responsivity were predictors of proximal responsivity. All 38 self-report items were included in the model as potential predictor variables with *p* < 0.1 as the entry and retention criteria. Given the developmental changes in feeding within the 6–18-month age range, child age in months was included as a control variable. 

The final regression model was used to compute scores for the responsive feeding questionnaire based on the predicted probability with items weighted differently depending on odds ratio values. To identify a cut point for the questionnaire to distinguish between dyads with distal vs. proximal responsivity, we selected the value that maximized the sum of sensitivity and specificity. Diagnostic validity statistics (sensitivity, specificity, positive predictive value, and negative predictive value) were calculated to assess the ability of the questionnaire to identify mother-child dyads with distal vs. proximal responsivity based on the video observation. Analyses were conducted using SAS version 9.4 (Cary, NC, USA). Given the exploratory nature of the project, we accepted *p* < 0.1

## 3. Results

We enrolled 67 mother-child dyads. Mothers had a mean age of 24.9 years with 1.8 children, including the study child (Table 1). Approximately half of the mothers (51%) had more than 5 years of education, including 14% who had completed secondary school and 9% with no schooling. Education was similar among fathers, 54% had more than 5 years of education, including 15% who had completed secondary school or beyond, and 6% with no schooling. Nearly all (98.5%) were Muslim. The mean monthly household income was equivalent to USD 166, the majority (76%) reported always or sometimes experiencing a monthly financial deficit. Approximately one-third (37%) of households were food secure, 21% were mildly food insecure, and 41% were moderately or severely food insecure. 

Children had a mean age of 12.8 months, 39% were females, 6% were underweight, 1 child was wasted, 15% were stunted, and none were overweight (Table 1). Almost all (96%) had been breastfed the previous day, 73% met the criterion required for minimum dietary diversity and 72% met the minimum meal frequency. Nearly all children consumed foods in the grains, rice, and tubers food group (98%). More than 50% of children consumed non-vitamin A rich fruits and vegetables, flesh foods, legumes/nuts/ and dairy. Nearly 50% (46%) of children consumed eggs and slightly more than one-third (37%) consumed fruits and vegetables high in vitamin A. 

To address the first objective, commonly reported feeding behaviors, we report maternal responses on the Responsive Feeding Questionnaire by quartiles (Table 2). The first quartile questions represent perceptions and behaviors that mothers reported rarely or almost never occurred, including mother threatening or speaking in a raised voice to encourage the child to eat, talking on the mobile during meals, unhappy or stressed during meals, mother and child eating together, and concern that the child eats too much or weighs too much. The fourth quartile questions represent perceptions and behaviors that mothers reported occurred most days, including confidence that the child eats enough food, positioning such that mothers and children can see one another, children communicating satiety, and mothers happy, talking to their child, and concerned that their child is not eating enough.

To address the second objective, comparison of responsive feeding observations with video-recorded feeding sessions, we found that based on the RCFCS video ratings, 29 (43%) of the mother-child dyads demonstrated distal responsivity and 38 (57%) demonstrated proximal responsivity. The stepwise logistic regression model fit well (AUC = 0.93) with nine items remaining in the final model (Table 3). Three questions had high odds of distal responsivity (Q2 pressure child to eat, Q13 child in a happy mood during meals, and Q29 mother positioned to see child’s face). Six questions had low odds of distal responsivity, indicating high odds of proximal responsivity (Q6 mother in a happy mood, Q10 child seated, Q17 mother talks to child, Q 23 wash child’s hands prior to meal, Q27 mother feels stress at meals, and Q33 mother concerned that child does not eat enough). 

Using the regression results, we computed scores for the Responsive Feeding Questionnaire and classified mother-child dyads into higher vs. lower odds of distal responsivity based on the optimal cut point for maximizing sensitivity and specificity. Table 4 shows the overlap between the responsivity classifications based on the RCFCS video ratings and the feeding questionnaire. The responsive feeding questionnaire demonstrated high diagnostic validity with sensitivity (90%), specificity (89%), positive predictive value (87%), and negative predictive value (92%). 

There were no differences between the proximal and distal categories by household or family demographics, children’s anthropometry, or dietary intake, with the exception that significantly more proximal group children consumed fruits and vegetables high in vitamin A compared to distal group children (47% vs. 24%, *p* = 0.051), (Table 1). 

## 4. Discussion

Our study sought to overcome the conceptual gaps and rethink responsive feeding by incorporating bidirectional mother-infant responsivity and early learning principles into the measurement and validation of a measure of responsive feeding. We described commonly reported feeding behaviors and then examined the concordance between caregiver-reported feeding behaviors and observations of video-recorded behaviors. Based on observations of mother-child dyads during a feeding episode, we differentiated distal verses proximal responsivity consistent with modulated patterns of mother-child responsivity that occur as young children are acquiring new skills; in this case feeding [15]. 

The responsive feeding questions that predicted distal responsivity (positioning the child to see the mother’s face, perceiving the child as happy, and pressuring the child to eat) highlight the relevance of considering the emotional context or tone of the meal. Distal responsivity requires mothers to step back from proximal interactions to enable their child to practice and build mastery of newly acquired skills without intrusion. Distal responsivity was characterized by mothers who perceived the child to be happy and positioned the child to see the mother, which facilitates modeling and checking in with the mother while eating. Although pressuring the child to eat appears to be counterintuitive to responsivity, it should be interpreted within the emotional tone of the meal. Pressuring occurred in a positive emotional tone, with no evidence of maternal stress or concerns about the child’s growth or dietary intake. Thus, questions about pressuring may have been interpreted by mothers as encouraging the child, marked by the child’s happy mood. If pressuring had occurred in the context of concerns about a child’s poor appetite or food refusal, it may be interpreted as coercive control or the caregiver’s attempt to control the child’s eating behavior [28]. Coercive control is typically used when caregivers lack confidence that their child can or will eat without additional pressure. By communicating the caregiver’s lack of trust in the child’s food regulatory ability, coercive control is an ineffective strategy and may set up a conflictual situation with the child [29]. Thus, feeding perceptions and behaviors, including responsivity, may be best considered within the emotional tone of the meal, rather than as individual behaviors [28]. Distal responsivity is often regarded as autonomy supportive, enabling children to practice acquired behaviors with the goal of achieving mastery [28]. Children whose mothers exhibit these behaviors may not elicit general responsivity behaviors because they are busy eating and do not want or need feedback or assistance. 

Proximal responsivity was associated with a mix of maternal-reported perceptions and behaviors, ranging from mothers feeling stressed and concerned that their child was not eating enough to talking and being in a happy mood while their child was eating. The finding that maternal concerns about children’s intake coupled with feelings of stress were associated with proximal responsivity is consistent with reports that child behaviors and maternal perceptions about children’s growth, health, and temperament drive maternal-child responsivity [4,30]. Mothers’ happy mood and talking during meals may reflect positive feelings of being engaged in their child’s feeding, even in the face of stress and concern. These findings illustrate how mothers’ perceptions regarding the child’s well-being are associated with their own behavior—a critical component of a reciprocal mother-child relationship during feeding. 

Children can begin to feed themselves by about 8 months. Thus, washing a child’s hands sets the stage for self-feeding—a component of autonomy promoting. Handwashing is a common feeding and eating behavior in Bangladesh, has successfully been introduced into feeding interventions [31], and may be particularly relevant in settings with poor hygienic conditions. Washing children’s hands before meals and having them seated during meals may facilitate children’ engagement and responsivity (and perhaps compliance) and may also elicit responsive behaviors from caregivers. 

Caregiver feeding practices vary across ethnicities and cultures [32,33], as well as by the family environment and by caregivers’ perceptions of their child’s health, appetite, and behavior. Thus, caregivers may adopt feeding behaviors designed to address specific goals for their child, such as increased consumption. Overall, the Bangladeshi mothers described daily feeding behaviors associated with nurturance and responsivity, including being in a happy mood, positioning to enable mother-child communication and modeling, talking to the child, clear communication from the child regarding satiety, and confidence that the child was eating enough food, although concerned that the child was not eating enough. These behaviors suggest that mothers are engaging in interactions with their child indicative of responsive feeding. Mothers’ concern that their child is not eating enough may be reflective of a country characterized by high rates of childhood morbidity and mortality related to malnutrition and infection [34]. This interpretation is partially supported by our finding that concerns about children eating or weighing too much were extremely rare, with no children meeting criteria for overweight. Bangladeshi mothers denied many harsh and non-responsive feeding practices, including threatening or yelling to encourage their child to eat, talking on a mobile phone, or being in an unhappy mood. They also denied eating together with their child, which may reflect the custom of mothers eating after children. 

Caregivers promote their children’s early development, including feeding, by establishing daily routines and expectations, and responding to children’s physical and emotional signals [28,35,36]. Children’s acquisition of regulatory processes, including eating, requires practice, which leads to mastery and feelings of competence and autonomy [15]. By modulating responsivity between proximal and distal support, caregivers facilitate children’s establishment of mastery through practice. This back-and-forth process results in a “goodness-of-fit” pattern, characterized by synchrony between caregivers’ expectations and feeding behavior and children’s eating and regulatory behavior. By embedding responsivity within a context of nurturance and support, children are primed to practice newly acquired behaviors and to build competence, confidence, and self-regulation related to eating [37]. These processes, referred to as scaffolding [15], have been linked to healthy dietary patterns and growth throughout life.

As with all studies, there are methodological weaknesses that should be considered. Our study was conducted in a single site by design, thereby limiting generalizability. Although nine items from the responsive feeding questionnaire demonstrated excellent sensitivity, specificity positive predictive value, and negative predictive value in predicting video-observed proximal and distal responsivity patterns, replication is necessary to determine whether these patterns are generalizable or specific to Bangladeshi mother-child dyads. The sample size was relatively small, though powered to detect a significant difference between proximal and distal responsivity. In spite of conducting feeding observations with a validated measure (RCFCS), including guidance from the developer and ongoing assessments of inter-observer reliability, we cannot rule out some degree of subjectivity in the ratings or guarantee homogeneity of caregiver-child feeding behavior across sites or conditions. 

A major contribution of our study is broadening the conceptualization of responsive feeding, comprised of proximal and distal responsivity, to recognize bidirectional caregiver-child patterns of responsivity, rather than unidirectional patterns of high versus low responsivity that have driven much of the previous research. In addition, the study included both maternal and child responsivity cues, observation coding based on a validated measure, high inter-observer reliability, and responsive feeding questions generated through a 2-phase Delphi procedure, validated within Bangladesh, and strong association with video-recorded observations of mother-child dyadic responsivity. 

Additional research is needed to differentiate distal responsivity from a lack of responsivity in which children receive either little guidance or non-responsive practices, such as coercive control. To determine discriminant and predictive validity of mother-reported responsive feeding perceptions and behaviors, future studies are necessary across cross-sectional samples that vary in health and nutritional status and longitudinal samples followed over time, respectively. Our findings promote greater understanding of the bidirectional, dyadic aspects of responsive feeding as well as the autonomy-promoting modulation between proximal and distal responsivity that enables infants and young children to acquire and practice healthy eating behaviors.

## 5. Conclusions

We identified nine maternal-reported items that differentiated between observation-defined proximal and distal responsivity among a sample of Bangladeshi mothers and their 6–18-month-old children. Proximal and distal responsivity vary as children are acquiring and practicing feeding skills and are interpreted within the emotional context of the meal, rather than through individual behaviors [28]. These initial steps lay the groundwork for the development of a simple yet valid and reliable measure for responsive feeding for global use.

## Figures and Tables

**Table 1:** Demographic and household characteristics of mother-infant dyads by proximal and distal responsivity.

Characteristics	Overall N = 67	Proximal Responsivity N = 38	Distal Responsivity N = 29	*p*-Value
**Mother**				
Maternal age (years), mean (SD)	24.9 (5.6)	25.5 (5.7)	24.2 (5.5)	0.342
Maternal education				
None, n (%)	6 (9.0)	4 (11)	2 (7)	0.949
1–5 years, n (%)	27 (40.3)	15 (39.5)	12 (39.4)	
6–8 years, n (%)	25 (37.2)	14 (36.5)	11 (39.6)	
Secondary School Certificate, n (%)	4 (6.0)	2 (5)	2 (7)	
Higher Secondary Certificate, n (%)	5 (7.5)	3 (8)	2 (7)	
Paternal education				
None	4 (6.0)	1 (2.6)	3 (10.3)	0.645
1–5 years, n (%)	27 (40.3)	19 (50.0)	8 (27.6)	
6–8 years, n (%)	20 (29.8)	8 (21.0)	12 (41.4)	
Secondary School Certificate, n (%)	6 (9.0)	3 (7.9)	3 (10.3)	
Higher Secondary Certificate, n (%)	10 (14.9)	7 (18.4)	3 (10.3)	
Religion				
Islam, n (%)	66 (98.5)	38 (100.0)	28 (96.6)	0.249
Hindu, n (%)	1 (1.4)	0 (0.0)	1 (3.5)	
Finances				
Total number of children mean (SD)	1.8 (0.9)	1.8 (0.9)	1.7 (0.9)	0.768
Monthly income, taka, mean (SD)	15410.5 (8081.0)	15697.4 (8133.6)	15034.5 (8139.3)	0.742
Monthly income, dollars, mean (SD) *	165.90 (87.51)	168.90 (87.51)	161.77 (87.95)	
Monthly Financial Deficit **				
Always, n (%)	13 (19.4)	6 (15.8)	7 (24.1)	0.665
Sometimes, n (%)	38 (56.7)	23 (60.5)	15 (51.7)	
Never, n (%)	9 (23.7)	7 (24.1)	16 (23.9)	
Food security ***				
Food secure, n (%)	25 (37.3)	15 (39.5)	10 (34.5)	0.994
Mildly food insecure, n (%)	14 (20.9)	7 (18.4)	4 (24.1)	
Moderately food insecure, n (%)	12 (17.9)	7 (18.4)	5 (17.2)	
Severely food insecure, n (%)	16 (23.9)	9 (23.7)	7 (24.1)	
**Child**				
Female, n (%)	26 (38.8)	13 (34.2)	13 (44.8)	0.377
Age (months), mean	12.8 (3.4)	12.5 (3.8)	13.2 (2.8)	0.440
Growth ****				
Weight-for-age z-score (WAZ), mean (SD)	−0.7 (0.9)	−0.8 (0.9)	−0.6 (0.8)	0.597
Length-for-age z-score (LAZ), mean (SD)	−1.2 (1.0)	−1.2 (1.0)	−1.1 (0.9)	0.612
Weight-for-length z-score (WLZ), mean (SD)	−0.2 (0.9)	−0.2 (1.0)	−0.1 (0.8)	0.897
Underweight (<−2 WAZ), n (%)	4 (6.0)	3 (7.9)	1 (3.5)	0.447
Stunted (<−2 LAZ), n (%)	10 (14.9)	7 (18.4)	3 (10.3)	0.358
Wasted (<−2 WLZ), n (%)	1 (1.5)	1 (2.6)	0 (0.0)	0.379
Diet during previous 24-h				
Breastfed, n (%)	64 (95.5)	36 (94.7)	28 (96.6)	0.722
Minimum dietary diversity, n (%) *****	49 (73.1)	28 (73.7)	21 (72.4)	0.908
Minimum meal frequency, n (%) ******	48 (71.6)	26 (68.4)	22 (72.4)	0.503
Food groups consumed, n (%)				
Grains, Rice, Tubers	66 (98.5)	38 (100.0)	28 (96.6)	0.249
Legumes/Nuts	39 (58.2)	20 (52.6)	19 (65.5)	0.289
Dairy	34 (50.8)	18 (47.4)	16 (55.2)	0.527
Flesh Foods	41 (61.2)	23 (60.5)	18 (62.1)	0.898
Eggs	31 (46.3)	17 (44.7)	14 (48.3)	0.774
Fruits & Vegetables high in Vitamin A	25 (37.3)	18 (47.4)	7 (24.1)	0.051
Other Fruits and vegetables	42 (62.7)	21 (55.3)	21 (72.4)	0.150

* Bangladesh taka converted to US dollars 0.0176 taka/US dollar. ** Monthly Financial Deficit as reported in Methods. *** Food Security Score was calculated using an adapted HFIAS Food Insecurity Scale: food secure (no items affirmed), mildly food insecure (compromised dietary quality or preferences as described in Methods. **** No children met criteria for overweight (WLZ > 2). ***** Minimum dietary diversity: 5 or more from 8 food groups in the previous 24 h. ****** Minimum meal frequency: Breast fed infants: age 6–8 months 2 or more meals per day; age 9–18 months 3 or more meals/day. Non-breastfed infants, age 6–18 months 4 or more meals and 2 milk feeds/day.

**Table 2:** Items, Mean Scores, and Quartiles in the Responsive Feeding Questionnaire.

Number	Item	Mean (SD) *
Quartile 1		
35.	How concerned are you that your child eats too much?	1.0 (0.2)
32.	How concerned are you that your child weighs too much?	1.1 (0.5)
20.	How often do you talk on the mobile phone during your child’s meals?	1.1 (0.3)
7.	How often do you eat a meal with your child?	1.3 (0.5)
8.	How often are you in an unhappy mood while your child is eating?	1.3 (0.6)
16.	How often do you threaten your child to get him/her to eat?	1.4 (0.7)
27.	How often do you feel hassled or stressed during meals with your child?	1.5 (0.9)
5.	How often do you raise your voice (e.g., eat eat) to get your child to eat?	1.6 (0.8)
25.	How often do you restrict your child’s arms during meals?	1.8 (1.1)
Quartile 2		
24.	How often do you hold your child in your lap during meals?	1.9 (1.1)
36.	How concerned are you about what your child eats?	1.9 (1.2)
30.	How often does your child eat meals at the same time every day?	1.9 (1.1)
10.	How often does your child stay seated during the meal?	1.9 (1.2)
9.	How often do you show your child that you are happy or unhappy with how your child is eating?	2.0 (1.2)
21.	How often do you encourage your child to touch the food during meals?	2.0 (1.0)
37.	How important is it for your child to finish all his/her food?	2.0 (1.1)
15.	How often do you promise to give your child something if he/she eats?	2.1 (1.1)
14.	How often does your child have a poor appetite during meals?	2.2 (0.9)
18.	How often is your child upset or distressed during meals?	2.2 (1.0)
22.	How often does your child feed him/herself at least one bite of food during meals?	2.2 (1.0)
Quartile 3		
2.	How often do you pressure your child to eat?	2.3 (1.2)
1.	How often does your child refuse to eat most of the meal?	2.5 (1.1)
19.	How often does your child have a good appetite during meals?	2.3 (0.8)
13.	How often is your child in a happy mood while he/she is eating?	2.4 (0.9)
3.	How often do you use a toy, TV, or mobile phone to get your child to eat?	2.4 (1.2)
4.	How often do you distract your child without a toy or other object (e.g., airplane game) to get him/her to eat?	2.5 (1.1)
11.	How often does your child let you know when he/she is hungry?	2.7 (1.0)
34.	How concerned are you about your child’s health?	2.7 (1.2)
Quartile 4		
23.	How often do you wash your child’s hands before meals?	2.8 (1.3)
31.	How concerned are you that your child does not weigh enough?	2.8 (1.3)
38.	How confident are you that your child eats enough food?	2.9 (1.0)
33.	How concerned are you that your child does not eat enough?	3.0 (1.1)
12.	How often does your child let you know when he/she is full?	3.0 (1.1)
6.	How often are you in a happy mood while you child is eating?	3.3 (0.7)
28.	How often do you feel happy during meals with your child?	3.3 (0.8)
26.	How often is your child positioned so he/she can see your face during meals?	3.3 (1.0)
29.	How often are you positioned so you can see your child’s face during meals?	3.6 (0.7)
17.	How often do you talk to your child during meals?	3.6 (0.7)

* Item scores range from 1 to 4. Items were recoded; higher values and higher quartiles indicate more often, greater concern, greater importance, or more confidence, as applicable.

**Table 3:** Logistic Regression Model Predicting Distal Responsivity.

Item	Variable	OR (95% CI)	*p*-Value	Distal vs. Proximal
Age	Infant age (months)	1.31 (0.96, 1.78)	0.089	
Q2	How often do you pressure your child to eat?	7.98 (2.22, 28.73)	0.002	Distal
Q6	How often are you in a happy mood while your child is eating?	0.28 (0.08, 1.01)	0.051	Proximal
Q10	How often does your child stay seated during the meal?	0.16 (0.05, 0.46)	<0.001	Proximal
Q13	How often is your child in a happy mood while he/she is eating?	5.36 (1.46, 19.64)	0.011	Distal
Q17	How often do you talk to your child during meals?	0.25 (0.06, 1.01)	0.051	Proximal
Q23	How often do you wash your child’s hands before meals?	0.29 (0.13, 0.67)	0.004	Proximal
Q27	How often do you feel hassled or stressed during meals with your child?	0.22 (0.07, 0.68)	0.008	Proximal
Q29	How often are you positioned so you can see your child’s face during meals?	13.11 (2.09, 82.41)	0.006	Distal
Q33	How concerned are you that your child does not eat enough?	0.15 (0.04, 0.58)	0.006	Proximal

Note: Odds ratios greater than 1 indicate higher odds of having distal responsivity; odds ratios less than 1 indicate lower odds of having distal responsivity. By design, lower odds of distal responsivity are interpreted as higher odds of proximal responsivity. Age was included as a control variable with the remaining variables entered in the model in a stepwise fashion using *p* < 0.1 as entry and retention criteria. Model AUC is 0.93.

**Table 4:** Number and Percentage of Mother-Infant Dyads by Observed and Predicted Distal and Proximal Responsivity.

Predicted (Self-Report Feeding Questionnaire)	Observed (Video Ratings)	
	Distal	Proximal
	n/N (Col %)	n/N (Col %)
Distal	26/29 (90%)	4/38 (11%)
Proximal	3/29 (10%)	34/38 (89%)

## Data Availability

The data presented in this study are available on request from the first or corresponding author. The data are not publicly available due to privacy issues.
